# Tumour-Associated Microangiopathic Haemolytic Anaemia with Thrombocytopenia: A Narrative Review and Case Study

**DOI:** 10.3390/jcm14072164

**Published:** 2025-03-22

**Authors:** Vedran Kovacic, Marijana Mikacic, Ivan Jerkovic, Tanja Ilic Begovic, Marina Maras

**Affiliations:** Internal Medicine Department, Division of Internistic Intensive Medicine with Clinical Pharmacology and Toxicology, University Hospital of Split, School of Medicine, University of Split, 21000 Split, Croatia

**Keywords:** microangiopathic haemolytic anaemia, thrombocytopenia, tumour

## Abstract

Thrombotic microangiopathy (TMA) is a category of diseases consisting of thrombocytopenia, microangiopathic haemolytic anaemia, and widespread occlusive microvascular thrombosis. We report two cases of a thrombotic microangiopathic syndrome associated with non-invasive mucinous cysts and mucinous adenocarcinoma. TMA was treated in both cases by surgical removal of the tumours. We hypothesise that mucin secretion in the case of non-invasive mucinous cysts and paraneoplastic secretion of antibodies in the case of mucinous adenocarcinomas are the causes of endothelial damage with thrombocytopenia and microangiopathic haemolytic anaemia. Finally, patients with TMA who exhibit unusual clinical characteristics or weak responses to plasma exchange should be examined for an underlying tumour. Tumour treatment is the preferred therapy for tumour-associated TMA.

## 1. Introduction

Thrombotic microangiopathy (TMA) is the collective term for a group of illnesses that include thrombocytopenia, occlusive microvascular thrombosis, and ischemic organ damage, most often in the kidneys and brain. TMA is the last common route for several clinical disorders. The two primary clinical entities that are a part of TMA syndromes are thrombotic thrombocytopenic purpura (TTP) and haemolytic uremic syndrome (HUS) [1]. TTP is the consequence of a deficiency of ADAMTS13 (von Willebrand factor-cleaving protease), and HUS is mediated by complement activation or Shiga toxin. There are several additional potential causes of TMA, including medications, infections, collagen vascular disorders, stem cell transplantation, and cancer. TMA syndromes (including Shiga toxin-mediated HUS, TTP, or atypical complement-mediated HUS) are characterised by microangiopathic haemolytic anaemia and thrombocytopenia [2]. Fever, neurological impairment, and renal failure are common clinical signs of TMA [3]. Renal failure is a common clinical characteristic of HUS, but neurological symptoms are more common in TTP. Intraluminal shearing mechanically destroys red blood cells, which manifests as Coombs-negative haemolytic anaemia with an increased schistocyte count, which is a result of erythrocytes passing through blocked arterioles with decreased lumen to “pin-point size”. Microvascular thrombi that obstruct blood flow and endothelial oedema are pathological characteristics. Some cases of thrombotic microangiopathy have been associated with malignant proliferation [4]. This paraneoplastic syndrome, known as cancer-associated TMA, is characterised by thrombocytopenia and Coombs-negative haemolytic anaemia with schistocytes. Malignancy-associated TMA is uncommon; one report [5] demonstrated that only 3.5% of patients had it, and another report [6] demonstrated that 7.8% of patients were first identified as having TTP before a systemic cancer was later found. Patients with TMA and disseminated malignancy reported longer symptom durations than those with TTP or HUS, had higher lactate dehydrogenase levels, were older, and more frequently did not respond to plasma exchange therapy [7]. TMA was infrequently correlated with benignant proliferation. We discuss two unique examples of TMA involving breast and pancreatic mucinous tumours.

## 2. Cases

### 2.1. First Case

**Figure 1:**
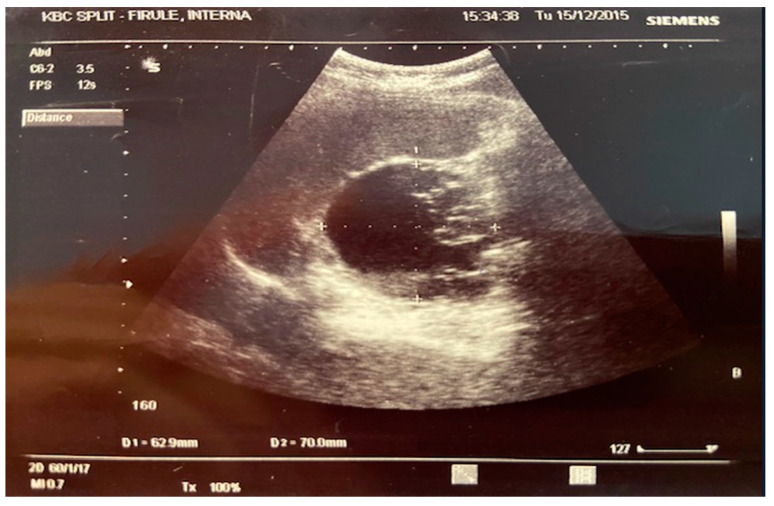
Ultrasound scan of the abdomen with findings of a cyst in the region of the pancreatic trunk.

A 27-year-old woman was admitted to the hospital with thrombotic macroangiopathic syndrome presented at admission with jaundice, pallor, dark urine output, tiredness, weakness, and bilateral lumbar pain without fever. She complained about those signs in the last few days before admission. She was taking medication for epilepsy chronically (lamotrigine). In her family, a case of malignant lymphoma was recorded (father of the patient). Clinical examination revealed only weakness, pallor, and jaundice, without purpura-like skin efflorescence or any neurologic disturbances. Laboratory evaluation at admission showed slight haemolytic anaemia with thrombocytopenia (platelet count 69 × 10^9^/L). The laboratory findings and their dynamics during hospitalisation are demonstrated in Table 1. Activated partial thromboplastin time and prothrombin time were normal. Plasma electrolyte levels were normal. Direct and indirect Coombs’ tests were negative. A peripheral blood smear study demonstrated the presence of schistocytes. As a pattern of microangiopathic haemolytic anaemia and thrombocytopenia was established, diagnosis of TTP was presumed, so immediately, plasma exchange therapy on a daily basis via central line was started, concomitant with systemic steroid therapy. In total, 17 sessions of plasma exchange were conducted, and subsequently, with variable success, platelet count and LDH were restored to the normal ranges; meanwhile, the process of haemolysis was partially stopped with consequent normalisation of haemoglobin concentration. Despite a good response, a series of peripheral blood smears revealed the constant presence of schistocytes. Concomitantly, a set of laboratory and imaging investigations were performed to potentially reveal the secondary aetiology of microangiopathic haemolytic anaemia and thrombocytopenia, as in our institution ADAMTS 13 plasma activity cannot be determined. Under usual circumstances, since it is not possible to determine ADAMTS 13 activity in our institution, we send the patient’s plasma sample (before plasmapheresis) to another institution to determine ADAMTS 13 activity (as well as ADAMTS13 inhibitors and antibodies). Unfortunately, for technical reasons, it was not possible to send the plasma sample before plasmapheresis in this case. The chest X-ray, laboratory panel for viruses (HBV, HCV, HIV), panel for immune system serology (ANA, anti-dsDNA, SS-A/Ro, AntiSS-B/La, ACA-CentB, aCL-IgM, aCL-IgG, PR3-ANCA, MPO-ANCA), oncomarkers (betaHCG, CA15-3, CA125, CEA, CIFRA21-1), electrophoresis of serum proteins, serum light chains kappa and lambda, and IHA for the *Echinococcus* antigen were in the normal ranges. Investigations of serum oncomarkers CA19-9 and NSE demonstrated higher values. Finally, an ultrasound scan of the abdomen found a cyst, sized about 80 mm in diameter with dense content within, placed in the region of the pancreatic trunk (Figure 1). A computed tomography (MSCT) abdominal scan confirmed this finding and revealed a cystic formation (sized 88 × 82 × 65 mm) in the region of the pancreatic trunk and tail, with just slight opacification of blood vessels. Finally, a magnetic resonance imaging (MRI) abdominal scan demonstrated the septated cystic formation with an irregular cyst wall. Despite the presence of schistocytes, we stopped plasma exchange sessions, and the patient was referred to an abdominal surgeon, and subsequently, distal pancreatotomy with splenectomy was performed. There was no evidence of the involvement of other abdominal organs. Finally, a histopathological examination of the received material proved this to be a noninvasive mucinous cyst of the pancreas with moderate-grade cell dysplasia (Neoplasma cysticum mucinosum pancreatis). After surgical removal of the cyst, repeated laboratory evaluations of the patient’s blood in the next 3 months were completely normal, with no detectable presence of schistocytes in blood smears. We concluded that the mucinous cyst of the pancreas was the source of endothelial damage with thrombotic microangiopathy (TMA) consecutively presented as microangiopathic haemolytic anaemia and thrombocytopenia, with no evidence of ischemic end-organ damage. The slower and more variable response to the therapeutic plasmapheresis emphasized this presumption.

### 2.2. Second Case

A 71-year-old previously healthy female was hospitalised in the intensive care unit due to weakness, fever, frontal headache, and dizziness, along with thrombocytopenia and anaemia. The day before admission, she had aphasia and left hemiparesis and was unconscious. A brain MSCT, cerebrospinal fluid, and an ultrasound of the blood vessels in the neck were not remarkable. A laboratory assessment upon admission revealed normal blood nitrogen components, thrombocytopenia (platelet count 35 × 10^9^/L), and severe haemolytic anaemia. The laboratory findings and their trends during treatment in the ICU are shown in Table 1. Microangiopathic haemolytic anaemia with thrombocytopenia was suggested by a low platelet count, Coombs-negative haemolytic anaemia, and the presence of schistocytes in peripheral blood smears. Therapeutic plasmapheresis procedures were started urgently on a daily basis, along with systemic corticosteroids. After an initial good response to plasmapheresis, a repeat episode of haemolysis with thrombocytopenia occurred, so rituximab was introduced. In total, the patient received nineteen therapeutic plasmaphereses and four doses of rituximab (700 mg per week). In the presence of an ADAMTS13 inhibitor factor, ADAMTS13 activity of less than 1% was confirmed. Antibodies of factor H or C1q, as well as the activation of the alternative complement pathway, were not demonstrated. At the same time, an extensive diagnostic workup was started. The MSCTs of the abdomen and thorax were normal. There was no malignant infiltration in the bone marrow biopsy specimen. On the ninth day of hospitalisation, the patient became respiratory distressed and was intubated, and mechanical ventilation was started. Her consciousness deteriorated to a coma, while her control brain MSCT remained normal. Finally, an ultrasound verified a node in the right breast. A plastic surgeon performed a tumourectomy of the right breast, and histopathological analysis confirmed mucinous adenocarcinoma. The patient’s platelet counts gradually normalised, and haemolysis stopped, along with recovery of the general condition and state of consciousness and the establishment of adequate verbal contact. After 5 days, the patient was weaned from mechanical ventilation and discharged in good general condition, without relapse of thrombotic microangiopathy during outpatient follow-up. The patient had thrombotic microangiopathic haemolytic anaemia, with neurological symptoms and reduced ADAMTS13 activity and the presence of anti-ADAMTS13 antibodies; thus, it was a classic form of immuno-mediated TTP. Therefore, treatment with plasmapheresis, corticosteroids, and rituximab was justified. However, the insufficient response to immunosuppressive therapy can probably be attributed to mucinous adenocarcinoma of the right breast. Namely, the patient’s clinical condition improved only after removal of the malignancy. Therefore, it can be speculatively proposed that the anti-ADAMTS13 antibodies in this patient were a paraneoplastic phenomenon.

## 3. Discussion

Here, we present examples of breast and pancreas mucinous tumour-associated TMA. This is a unique report of TMA correlated with a non-invasive cystic pancreatic mucinous tumour, as well as the first report of breast cancer-associated TMA with the presence of an inhibitor of ADAMTS13.

TMA (thrombotic microangiopathy) is a kind of microvascular thrombosis with some degree of vessel lumen occlusion that occurs concurrently with multiorgan failure and is frequently accompanied by symptoms of the nervous system, kidneys, or heart. The diagnosis of thrombotic microangiopathies, including TTP, requires the presence of thrombocytopenia with nonimmune microangiopathic haemolytic anaemia (schistocytes found as erythrocytes fragments, reduced haptoglobin concentration, elevated reticulocyte count, LDH, and indirect bilirubin [8]).

Von Willebrand factor (VWf) plays a crucial role in the development of TTP. VWf is produced in endothelial cells in the blood, where it normally appears as large multimers that are split into smaller multimers during circulation by a VWf-cleaving metalloproteinase named ADAMTS13. An ADAMTS13 deficiency leads to the accumulation of abnormally large VWf multimers, which causes TTP: microvascular thrombi with platelet agglutination [9]. In the congenital form of TTP, inborn ADAMTS13 alterations are discovered, leading to an inability to cleave large VWf multimers, which accumulate sequentially and cause platelet aggregation with microthrombosis. Anti-ADAMTS13 antibodies, cancer (and cancer therapy), chronic inflammation, pregnancy, liver disease, and disseminated intravascular coagulation are all associated with acquired ADAMTS13 deficiencies. In acquired or congenital TTP, ADAMTS13 activity is frequently less than 5% of normal activity [5]. A severe ADAMTS13 deficit (5% of normal) has been postulated as the primary pathogenic component of TTP [10]. Higher ADAMTS13 activity has been documented in malignant patients (median value 50%, range 13–100%) [7].

In our second case, ADAMTS13 activity was less than 1%, and ADAMTS13 inhibitors were present, so we assumed that the mechanism of TMA formation was related to the paraneoplastic production of antibodies against ADAMTS13. This assumption was supported by significant neurological symptoms in this patient, which are not common in TMA associated with tumours, despite classic anti-ADAMTS-mediated TTP. Breast cancer-associated TMA is almost always caused by bone marrow infiltration (which was negative in the reported case), highlighting the unusual mechanism of TMA in the case.

TMA has been linked to a variety of cancers, and so far, a whole series of malignant diseases that have been associated with microangiopathic haemolytic anaemia have been described in the literature [11] (Table 2).

A category of diseases known as cancer-associated thrombotic microangiopathy includes thrombocytopenia, microvascular thrombosis, and perhaps ischemic end-organ damage. Except for prostate cancer, where atypical HUS is a common presentation, serious microangiopathic haemolytic anaemia and thrombocytopenia can be associated with systemic metastatic malignancy and may be misdiagnosed as TTP [12].

Breast, lung, and stomach cancer have been the most prevalent cancer-related TMA cases recorded [13], especially in the case of metastatic illness [14,15], and in advanced mucin-producing adenocarcinomas [16,17]. Lechner et al. reported TMA linked to 154 cases of solid cancer (gastric, breast, prostate, and lung) and to 14 cases of lymphoma (Hodgkin disease, angiotropic lymphoma, diffuse large cell lymphoma, and myeloma) [12]. The most prevalent histological subtype in individuals with cancer-associated TMA is adenocarcinoma; 91.8% of the malignancies are metastatic, and 19.4% are solid tumours. The most prevalent kind is gastric carcinoma (26.2%), followed by breast (21.4%), prostate (13.7%), and lung cancer (9.5%). In the vast majority (81.1%) of patients, a bone marrow biopsy or autopsy is performed to confirm bone marrow infiltration with cancer cells. Occasionally, bone marrow necrosis or fibrosis is related to bone marrow infiltration. In a study of 51 patients with TMA related to breast cancer, 71% exhibited bone marrow invasion [18]. The majority of cancer patients had ADAMTS13 levels of more than 20%.

The majority of cancer-related TMA cases have been recorded in patients with mucin-producing adenocarcinomas and disseminated malignancies, particularly with tumour invasion of the bone marrow. Therefore, it is definitely necessary to include diagnostic bone marrow examination (preferably biopsy) in the diagnostic panel of patients with microangiopathic anaemia, as demonstrated in our second case. A study by Lohrmann et al. reported that TMA was found in 5.7% of individuals with metastatic cancer [19]. A case of TMA was also demonstrated in multiple endocrine neoplasia type I with pituitary adenoma, bronchial carcinoid with liver metastases, and adrenal adenoma [20].

The pathophysiology of TMA associated with malignancy is poorly known [21] and distinct from the classic TTP pathogenesis. Chemotherapy-induced endothelial damage, angiogenesis, systemic microvascular metastases, tumour development, bone marrow necrosis or metastasis, secondary myelofibrosis, and angiogenesis are all possible mechanisms for the formation of cancer-associated TMA [22,23]. Cancer-associated TMA is primarily caused by tumour invasion, which causes endothelial cell damage in bone marrow vessels. Endothelial damage processes in bone marrow may be linked to aberrant angiogenesis, aggressive tumour development, and secondary myelofibrosis [24]. It has also been documented that TMA linked to bone marrow necrosis (myeloid tissue and medullary stroma necrosis with bone preservation) is the first sign of lung cancer [25] or the earliest sign of widespread colon cancer [26]. In metastatic carcinoma, bone marrow necrosis may contain cancer cell aggregates and vascular mechanical obstruction [27]. Cancer-related TMA is most frequent in subjects with known metastatic illness, although it can also occur in patients with non-metastatic cancer or even occult cancer. A bone marrow biopsy is recommended if cancer-induced TMA is suspected. The presence of cancerous cells in the bone marrow validates the diagnosis.

Endothelial damage caused by tumour cell emboli is another proposed mechanism of cancer-associated TMA, with enhanced endothelial cell release of ultra-large VWf multimers and subsequent platelet aggregation and red blood cell disintegration owing to interaction with intraluminal thrombi [28]. This is referred to as diffuse microscopic pulmonary involvement [29]. Microthrombi in cardiac arterioles and capillaries have also been seen [20]. A case of carcinocythemia with thrombotic microangiopathy and metastatic breast cancer was also presented, and direct interactions between circulating carcinoma cells and erythrocytes, as well as tumour emboli with fibrin thrombi in tiny blood vessels, were potential causes of TMA [30]. In addition, microangiopathic thrombocytopenia, haemolytic anaemia, and microvascular clusters of breast cancer cells were seen in the lungs, bone marrow, and other organs [31]. Possibly causing factors of TMA in these situations include systemic arteriolar and capillary blockage by tumour cells, resulting in the fragmentation of red blood cells and possible platelet consumption in tumour emboli at high shear rates [32].

Chemotherapy-induced TMA could be antibody-mediated or dose-dependent [33].

ADAMTS13 activity and VWf multimers are generally normal in most cancer-associated TMA (the median value of ADAMTS13 activity is 50% in those with cancer-associated TMA) [34]. According to one study, only three of eight patients with TMA associated with disseminated malignancy had a decreased level of ADAMTS13 [21]. Additionally, ADAMTS 13 antibodies were found in several cases of lymphoma-associated TMA [12].

Mucin’s influence on endothelial dysfunction can lead to the production of large VWf multimers. Cancer-associated TMA has been detected often in mucin-producing adenocarcinomas (lung, colon, stomach, and breast) [35].

Noninvasive tumours are rarely related to TMA. It was found that a prolactin-secreting pituitary adenoma was associated with TMA (confirmed as von Willebrand factor-positive microthrombi in the arterioles and capillaries of several organs, primarily the heart and brain) [36]. TMA can be exacerbated by high prolactin levels [37]. A case of a benign pelvic tumour, recurrent pulmonary emboli, and microangiopathic haemolytic anaemia without thrombocytopenia was documented [38]. Similarly, a patient with a borderline serous tumour was found to have microangiopathic haemolytic anaemia and thrombocytopenia [39].

Our first case is a unique report of a benign pancreatic cystic lesion connected to TMA. We postulated that the primary risk factor for the development of TMA was the cyst’s mucinous nature in our first patient. In cases of solid cancer and TMA, only mild signs of cerebral dysfunction were demonstrated [12]. Our first case did not show signs of cerebral dysfunction, although the second case had remarkable neurological abnormalities.

Plasma exchange therapy is still the preferred course of treatment for classic TTP in order to eliminate VWf multimers and potential ADAMTS13 autoantibodies. Plasma exchange should start when thrombocytopenia and microangiopathic anaemia are present [40]. Despite the excellent outcomes of plasma exchange in the treatment of TTP, its role in cancer-associated TMA has to be reconsidered [41]. Plasma exchange treatment and immunosuppression therapy often have little success with TMA associated with tumours [42]. One exception is TMA associated with prostate cancer, which showed a positive response to plasma exchange.

In our cases, only surgical excision of the tumours entirely cured the concerning course of microangiopathic anaemia. The most essential care approaches for cancer-associated TMA are a surgical treatment of the underlying tumour or early initiation of suitable chemotherapy for systemic malignancy with avoidance of medications directly related to TMA [43].

In patients with any sort of TMA, platelet transfusion might cause severe exacerbations due to the increased formation of microvascular thrombi and it should be avoided. As a result, platelet transfusion is not a suitable treatment option for tumour-associated TMA [44].

Finally, individuals with TMA who do not respond to plasma exchange should be evaluated for an underlying tumour (including a bone marrow biopsy). Because plasma exchange is associated with serious consequences, it is best to avoid it in situations with known malignancy [45].

Secondary TMA could be identified by normal plasma ADAMTS13 activity. TTP is defined by a severe ADAMTS13 deficiency (activity of 10%). Higher LDH levels, advanced age, coagulation problems, and poor liver function all point to cancer-related TMA rather than TTP. In our second case, we demonstrated a patient with low ADAMTS13 activity and anti-ADAMTS13 antibodies with proven breast adenocarcinoma, so vigorously searching for possible tumours should be performed even in cases with low ADAMTS13.

Treatment of the underlying tumours should mitigate the ominous clinical course of TMA, although disseminated cancer-induced TMA has a very bad prognosis. A group of disseminated cancer-induced TMA reported a median survival of only 3 days, and a cohort analysis reported a mortality rate of nearly 50% within 1 month of diagnosis [46]. On the contrary, surgical treatment could stop haemolysis in cases of solid tumours associated with TMA.

## 4. Conclusions

Thrombotic microangiopathy (TMA) can be a consequence of malignant tumours, especially mucinous adenocarcinomas. Rarely, benign tumours can lead to TMA. Patients with TMA who exhibit unusual clinical characteristics or weak responses to plasma exchange should be examined for an underlying tumour. In patients with microangiopathic anaemia of unclear aetiology, it is necessary to include diagnostic bone marrow workup. Tumour treatment is the preferred therapy for tumour-associated TMA.

## Figures and Tables

**Table 1:** The laboratory findings and their dynamics during hospitalisation in both presented cases.

**First Case**	**Haemoglobin Level (g/L)**	**Reticulocyte Count (Percents with 10^9^/L)**	**Platelet Count (10^9^/L)**	**Serum Haptoglobin (g/L)**	**Lactate Dehydrogenase (U/L)**	**Total Bilirubin (µmol/L)**	**Aspartate Aminotransferase (AST) (U/L)**	**Alanine Aminotransferase (ALT) (U/L)**	**Creatinine (µmol/L** **)**
1. day	111	14.0 (458)	69	<0.1	1993	116	95	29	82
6. day	96	3.2 (105)	144	0.15	118	7	54	32	94
15. day	89	4.1 (123)	126	1.17	127	5	45	29	83
21. day	80	3.2 (89)	81	1.35	123	5	59	28	67
30. day	93	2.3 (76)	171	1.32	95	6	49	31	66
**Second Case**									
1. day	71	19.0 (540)	35	<0.1	1128	84	42	33	63
3. day	69	25.0 (740)	26	0.09	1297	79	56	32	64
5. day	78	25.6 (720)	21	0.12	1249	45	78	61	54
7. day	82	25.8 (737)	44	1.49	750	29	57	39	122
10. day	93	18.3 (438)	10	0.08	1615	53	84	65	62
20. day	106	1.9 (61)	102	2.52	410	22	24	50	56
30. day	127	1.1 (31)	211	2.32	386	24	24	44	45

**Table 2:** Types of malignant diseases associated with cases of microangiopathic haemolytic anaemia (adapted from [11]).

Gastrointestinal Malignancies	
	Stomach Cancer Colon cancer
	Anal canal squamous cell carcinoma
	Metastatic appendicular carcinoma
Breast cancer	
Lung cancer	Adenocarcinoma
	Squamous cell carcinoma
	Small cell lung cancer
Genitourinary malignancies	Renal cell carcinoma
	Seminal vesicle tumour
	Prostatic cancer
	Ovarian cancer
Hepatobiliary cancers	Hepatobiliary malignancies
	Hepatocellular carcinoma
	Pancreatic cancer
	Cholangiocarcinoma
Endocrinologic cancers	Multiple endocrine neoplasia type 1
	Pheochromocytoma
	Neuroendocrine tumour
	Prolactin-producing pituitary adenoma
	Bronchial carcinoid with liver metastases
	Multiple endocrine neoplasia type I with pituitary adenoma
Haemathologic malignancies	Acute lymphoblastic leukaemia
	Multiple myeloma
	Non-Hodgkin lymphoma
	Myelodysplastic syndrome
Others	Kaposi sarcoma
	Cardinom of unknown origin
	Adrenal adenoma

## Data Availability

The data presented in this study are available on request from the corresponding author.
